# Time Course of Recovery Following CrossFit^®^ Karen Benchmark Workout in Trained Men

**DOI:** 10.3389/fphys.2022.899652

**Published:** 2022-08-19

**Authors:** Ivo Vieira de Sousa Neto, Nuno Manuel Frade de Sousa, Frederico Ribeiro Neto, Joao Henrique Falk Neto, Ramires Alsamir Tibana

**Affiliations:** ^1^ Laboratory of Molecular Analysis, Graduate Program of Sciences and Technology of Health, University of Brasilia, Brasilia, Brazil; ^2^ Laboratory of Exercise Physiology, Faculty Estacio of Vitoria, Vitoria, Brazil; ^3^ Paralympic Sports Program, SARAH Network of Rehabilitation Hospitals/SARAH Brasilia, Brasilia, Brazil; ^4^ Athlete Health Lab, Van Vliet Complex, Faculty of Kinesiology, Sport and Recreation, University of Alberta, Edmonton, AB, Canada; ^5^ Graduate Program in Health Sciences, Faculty of Medicine, Federal University of Mato Grosso (UFMT), Cuiabá, Brazil

**Keywords:** functional fitness, high intensity functional training, periodization, overreaching, muscle recovery

## Abstract

The establishment of fatigue following the acute exercise stimulus is a complex and multi-factorial process, that might arise due to a range of distinct physiological mechanisms. However, a practical method of assessing CrossFit^®^ athletes’ recovery status has been neglected entirely in real-world sporting practice. The study describes the acute and delayed time course of recovery following the CrossFit^®^ Benchmark Workout Karen. Eight trained men (28.4 ± 6.4 years; 1RM back squat 139.1 ± 26.0 kg) undertook the Karen protocol. The protocol consists of 150 Wall Balls (9 kg), aiming to hit a target 3 m high. Countermovement jump height (CMJ), creatine kinase (CK), and perceived recovery status scale (PRS) (general, lower and upper limbs) were assessed pre, post-0h, 24, 48 and 72 h after the session. The creatine kinase concentration 24 h after was higher than pre-exercise (338.4 U/L vs. 143.3 U/L; *p* = 0.040). At 48h and 72 h following exercise, CK concentration had returned to baseline levels (*p* > 0.05). The general, lower and upper limbs PRS scores were lower in the 24-h post-exercise compared to pre-exercise (general PRS: 4.7 ± 1.5 and 7.7 ± 1.7; *p* = 0.013; upper limbs PRS: 6.6 ± 1.3 and 7.5 ± 1.3; *p* = 0.037; lower limbs PRS: 3.9 ± 2.5 and 7.3 ± 0.1; *p* = 0.046). Our findings provide insights into the fatigue profile and recovery in acute CrossFit^®^ and can be useful to coaches and practitioners when planning training programs. Moreover, recovery status can be useful to optimize training monitoring and to minimize the potential detrimental effects associated with the performance of repeated high-intensity sessions of CrossFit^®^.

## Introduction

CrossFit^®^ training programs are usually characterized by a high training intensity, with most of the sessions being performed at high intensities (Meyer et al., 2017). The training sessions contemplate the development of multiple physical abilities, through the use of different exercises such as weightlifting exercises (clean and jerk, snatch, and its variations), powerlifting (bench press, overhead press, deadlift, front, and back squat), and metabolic conditioning (Claudino et al., 2018; Martinez-Gomez et al., 2020). A recent systematic review identified that CrossFit^®^ training sessions normally cause a substantial metabolic stress, leading to metabolite accumulation (e.g., lactate up to 18 mmol/L), and to high levels of fatigue, impairing the ability to repeat the initial performance in a countermovement jump, a potential indicator of neuromuscular fatigue, are also seen immediately after the sessions. These effects may last up to 48 h, depending on the characteristics of the session performed (Claudino et al., 2017; Cooper et al., 2020). In addition, the high number of repetitions performed, often to the point of muscular failure, increase markers of exercise-induced muscle damage (interleukin-6 - IL-6, and creatine kinase—CK), with these concentrations remaining elevated up to 24 h post-exercise (Claudino et al., 2018).

When comparing the perceptual responses and post-exercise physical disfunction between a CrossFit^®^ session and a session based on the guidelines of the American College of Sports Medicine, Drum et al. (2017) found significant differences between sessions. CrossFit^®^ participants reported a higher rating of perceived exertion (RPE) and a greater perceived number of hard training days per week. Also, feelings of excessive fatigue, muscle soreness, muscle swelling, shortness of breath, muscle pain to light touch, and limited movement in muscles used during exercise within 48-h post-exercise were also higher in CrossFit^®^ participants. However, these responses were observed in a cross-sectional study, which limits the understanding of the cause-effect relationship (Wang and Cheng, 2020) that exists between a specific CrossFit^®^ Workout session and physiological outcomes. Since adaptations caused by exercise training may result from the temporal summation of acute responses (Rockl et al., 2008), understanding the role of recovery status in a time-dependent manner is first to step to understand fatigue status. Comprehending the time-course of recovery following CrossFit^®^ session is important for minimizing the risk of maladaptation due to insufficient recovery between each stimulus and might assist in ensuring optimal exercise monitoring.

The development of fatigue following the individual’s physiological and perceptual responses to a stimulus, is a complex and multi-faceted phenomenon, that might arise due to a variety of different mechanisms (Halson, 2014). Recovery, therefore, is also a multifactorial process, and as such, the assessment of the recovery–fatigue continuum should be relative to the demands of the sport or activity performed (Kellmann et al., 2018). While performance measures represent the most sport-specific outcomes, other physiological and psychological measures provide integral information on an athlete’s recovery (Kellmann et al., 2018). Stress markers such as creatine kinase (CK) counter movement jump (CMJ) and perceived recovery status (PRS) remain largely unknown in CrossFit^®^ training programs, despite their potential to identify athletes’ recovery status following exhaustive sessions (Tibana et al. 2019).

Despite the importance of performance and physiological markers, an athlete’s perception of their “readiness to perform” can also be described as a critical determinant of recovery. In this context, Laurent et al. (2011) proposed a “Perceived Recovery Status” (PRS) scale, which is similar but opposite to a perceived exertion scale (RPE) (10–12). Both scales are based on the psychophysiological status of the athlete. However, while the rating of perceived exertion (RPE) is utilized during or after a session, the PRS scale is utilized prior to the session to identify the athletes’ recovery status. The PRS scale has been shown to be a reliable tool to assess the perceived recovery state of individuals, demonstrating accuracy (>80%) in identifying changes in performance when the individuals reported feelings of being under-recovered (Laurent et al., 2011). A practical method of assessing athletes’ recovery status prior to a session might allow coaches and practitioners to adjust the training session to match the individuals’ current recovery status, potentially optimizing training outcomes (Laurent et al., 2011; Sikorski et al., 2013).

Thus, the purpose of this study is to describe the acute and delayed time course of recovery following the CrossFit^®^ benchmark workout Karen in healthy trained subjects. The development of fatigue following the individual’s physiological and perceptual responses to a stimulus, is a complex and multi-faceted phenomenon, that might arise due to a variety of different mechanisms it was hypothesized that the PRS scale would provide an accurate assessment of the participants’ recovery status, and that this would be mirrored by the changes in CK and muscle performance, assessed via a countermovement jump (CMJ). This variety of tools to monitor recovery are practical for daily use due to low cost and time accompanied by simple interpretations.

## Materials and Methods

### Participants

Eight male subjects (age 28.4 ± 6.4 years old; 1RM back squat: 139.1 ± 26.0 kg) were recruited. All participants were free of injury and known illnesses, were not using drugs to enhance performance, and had a minimum experience of 6 months with CrossFit^®^ and were familiar with all exercises used in the study. The subjects trained five times a week, each training session consisting of approximately 10 min of warm-up, 40 min of strength and power training, and 20 min of metabolic conditioning. Indirect maximal aerobic capacity (VO_2_ max), assessed via a maximal 2-km rowing test (Klusiewicz et al., 2016; Tibana et al., 2021) and strength (1RM) are described in Table 1, and were assessed 2 weeks before the participants completed the testing protocol. Participants performed one repetition maximum (1 RM) test for back squat according to procedures recommended by the National Strength and Conditioning Association (Lloyd et al., 2016). During this exercise period, standard instructions regarding the procedures of the test protocols and the appropriate execution of the exercise technique were supplied by an experienced investigator (Tibana et al., 2021). The participants were advised to refrain from ingesting alcohol in the 24 h before any of the tests, to avoid exercise in the 48 h before the protocol and in the 72 h after the workout of the day (WOD), and to maintain their normal daily diet and hydration during the study. All participants signed an informed consent document, and the study was approved by the University Research Ethics Committee for Human Use (2.698.225; 7 June 2018) and conformed to the Helsinki Declaration on the use of human participants for research.

**TABLE 1:** Baseline sample demographics and performance characteristics (*n* = 8).

Variables	Mean ± SD
Age (years)	28.4 ± 6.4
Body mass (kg)	80.4 ± 4.9
Height (m)	1.8 ± 0.1
VO2 (ml/kg/min)	53.6 ± 3.5
Maximal Rowing test 2 km (sec)	447.3 ± 17.1
Back squat (kg)	139.1 ± 26.0
Back squat rel (kg/kg)	1.5 ± 0.7
Karen (sec)	613.8 ± 115.0

Note: Variables are expressed as mean and standard deviation (±). Rel: relative (back squat/body mass).

### Experimental Design

**FIGURE 1:**
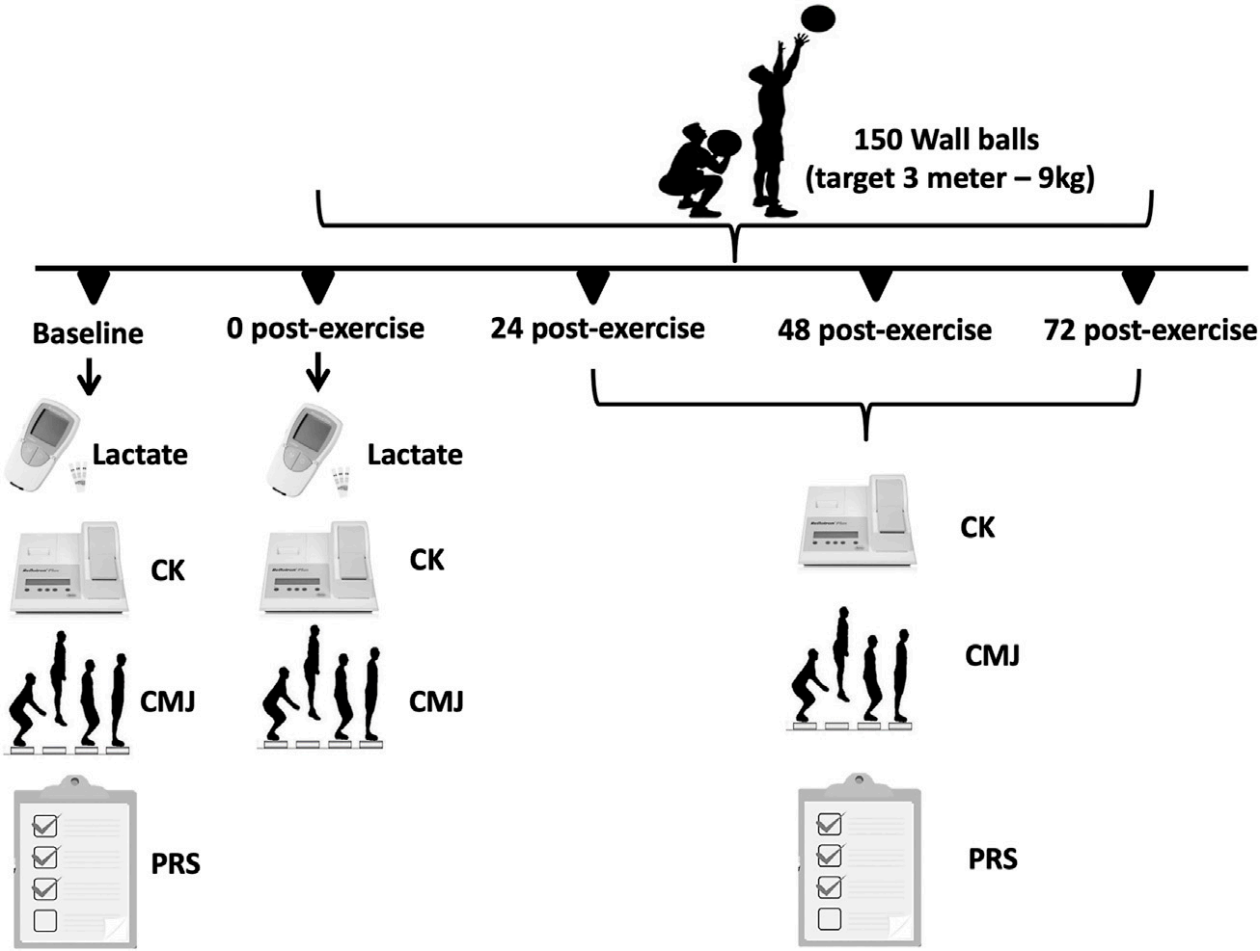
Schematic study design and timeline used to examine the time-course effects of creatine kinase, countermovement jump performance and the PRS scale.

This study was designed to analyze the time-course of recovery of physiological, psychological and performance responses in trained adult men, following the completion of the CrossFit^®^ benchmark workout Karen. The protocol consists of 150 repetitions of wall balls, with athletes aiming to hit a target 3 m high, using a 9 kg medicine ball. All participants were experienced with the protocol, having previously performed it a minimum of 4 times as part of their own training. Each participant performed the session individually. In this study, the benchmark Karen was the independent variable, while the dependent variables consisted of changes in creatine kinase, countermovement jump and PRS scale (general, lower, and upper limbs) (Figure 1).

### Karen Protocol

The CrossFit^®^ WOD Karen corresponds to a timed protocol that utilizes one element (medicine ball throws; 9.07 kg for a height of 3 m). The aim is to complete the task of performing 150 medicine ball throws to a wall in the shortest time possible. Therefore, a better performance in this WOD is indicated by a shorter time to complete the protocol. The Karen protocol was chosen because it consists of only one exercise and because of the large number of repetitions performed as fast as possible. Also, Karen protocol is very popular and extremely usual among the WOD routines.

### Creatine Kinase and Blood Lactate Analysis

Whole-blood creatine kinase activity was assessed from a single fingertip capillary sample with the subject in a seated position. After pre-warming the hand, a sample of blood (30 μL) was obtained and analyzed using a colorimetric assay procedure (Reflotron, Boehringer Mannheim, Germany). Before each testing session, quality control (calibration) measurements were undertaken according to the manufacturer’s recommendations. The ‘‘normal’’ reference range for creatine kinase activity, as provided by the manufacturer, is 24–195 U/L.

The blood lactate collection, management, and analysis were determined according to Falk Neto et al. (2020). Capillary blood samples were collected through a transcutaneous puncture on the medial side of the tip of the middle finger using a disposable hypodermic lancet (Falk Neto et al., 2020). Blood lactate concentration was determined by photometric reflectance on a validated Portable Accutrend Plus system (Roche, Sao Paulo, Brazil).

### Perceived Recovery Scale

**FIGURE 2:**
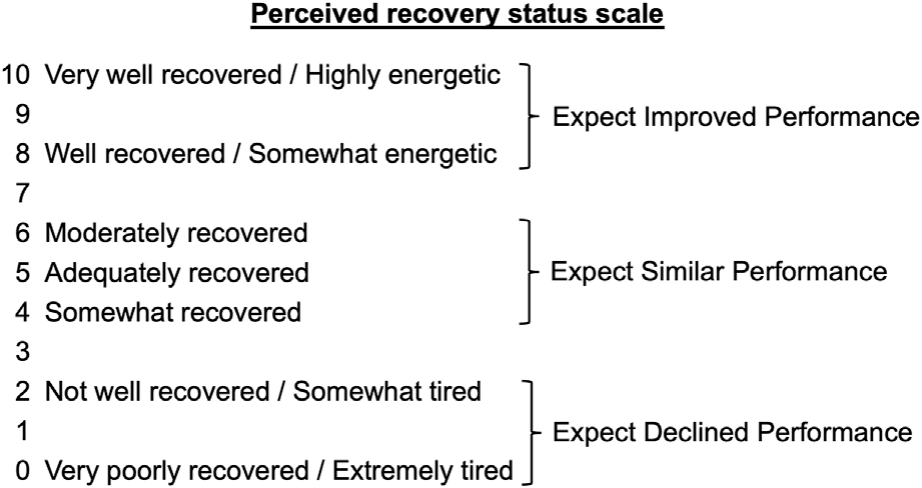
The PRS Scale according to Laurent et al. (Laurent et al., 2011).

Immediately before the training sessions, the athletes were asked to rate their recovery status according to the PRS Scale. The scale (Figure 2) ranges from 0 to 10, with a score of “0” indicating that the athlete is “very poorly recovered/extremely tired” and a score of “10” indicating that the athlete is “very well recovered/highly energetic”. A score of 0, 1, or 2, is associated with an expected reduction in performance, while a score of 8, 9, or 10, means an improvement in performance is expected. The range of values between three and seven indicate that no changes in performance are expected (Laurent et al., 2011).

### Countermovement Jump Height

For the CMJ height, a jump platform (Jump System 1.0, Cefise Ltda.) was used. The athlete was positioned, barefoot, in the interior of the platform, with their hands fixed at their waist. The test consisted of performing a maximal vertical jump. The athletes were instructed to swing their arms back and aim to jump as high as possible while using the momentum created with their movement. Two jumps were performed with a 1-min interval between them. The participants’ highest jump (in centimeters) was considered as the maximal CMJ height and utilized for subsequent analyses (Haugen et al., 2020). The CMJ was chosen because is a simple, practical, valid, and very reliable measure of lower-body power. The CMJ has been shown to be the most reliable measure of lower-body power compared to other jump tests (Petrigna et al., 2019).

### Statistical Analysis

The data are expressed as mean value ±standard deviation (SD). Shapiro–Wilk test was used to check for normal distribution of study variables (all variables presented normal distribution). Paired sample *t*-test was used to compare blood lactate concentration and RPE pre- and post-exercise session. Cohen’s *d* effect size (ES) was calculated to verify the magnitude of the difference between pre-test, and post-test. The ES are classified as: trivial (*d* lower than 0.10); small (*d* between 0.10 and 0.29); moderate (*d* between 0.30 and 0.49); large (*d* between 0.50 and 0.69); very large (*d* between 0.70 and 0.89), and perfect (*d* of 0.90 or greater). A repeated measures ANOVA was used to compare CK, PRS and CMJ between pre- and post-exercise session (24, 48, and 72 h after exercise session). Repeated measures ANOVA was also used to compare the score between general, upper and lower limbs of PRS scale. Compound sphericity was verified by the Mauchley test. When the assumption of sphericity was not met, the significance of F-ratios was adjusted according to the Greenhouse–Geisser procedure. Tukey’s post-hoc test with Bonferroni adjustment was applied in the event of significance. Cohen’s *f* effect size (ES) for ANOVA was calculated to estimates the proportion of variance in the present sample. The Cohen’s *f* effect size is classified as: small (*f* = 0.10); medium (*f* = 0.25); large (*f* = 0.40). The power of the sample size (1—ß) was determined using post hoc analysis on G*Power version 3.1.9 (Faul et al., 2007) and it is presented in the results section for each analysis. The Pearson correlation was used to evaluate correlations between PRS, CK and CMJ (pre-test, 24, 48, and 72 h post-session values grouped). The magnitude of the correlations was classified as: *r* ≤ 0.1 trivial; 0.1 < *r* ≤ 0.3 small; 0.3 < *r* ≤ 0.5 moderate; 0.5 < *r* ≤ 0.7 large; 0.7 < *r* ≤ 0.9 very large; > 0.9 almost perfect (Hopkins et al., 2009). The level of significance was *p* ≤ 0.05 and SPSS version 20.0 (Somers, NY, United States) software was used.

## Results

### Completion Time

The average time to complete the 150 repetitions of wall ball was 597 ± 111.6 s. The fastest volunteer completed the exercise session in 495.6 s and the slowest in 795 s.

### Physiological, Biochemical, and Neuromuscular Responses

The blood lactate concentration and RPE presented a statistically significant increase after the exercise session (blood lactate concentration, pre: 3.0 ± 0.7 mmol/L and post: 17.5 ± 3.0 mmol/L, *p* ≤ 0.005; ES = 4.63; RPE, pre: 1.6 ± 0.5 and post: 9.0 ± 0.8 mmol/L, *p* ≤ 0.005; ES = 10.59).

**FIGURE 3:**
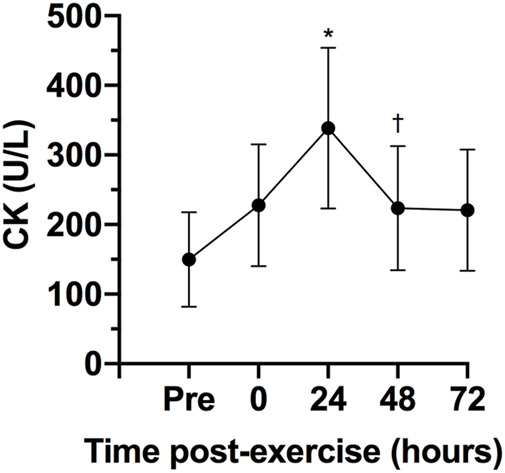
Variables are expressed as mean and standard deviation (±). Creatine kinase concentration (CK) during pre-test, post-test, 24, 48 and 72 h post-test. *p ≤ 0.05 for pre-exercise; †*p* ≤ 0.05 for 24-h post-exercise.

**FIGURE 4:**
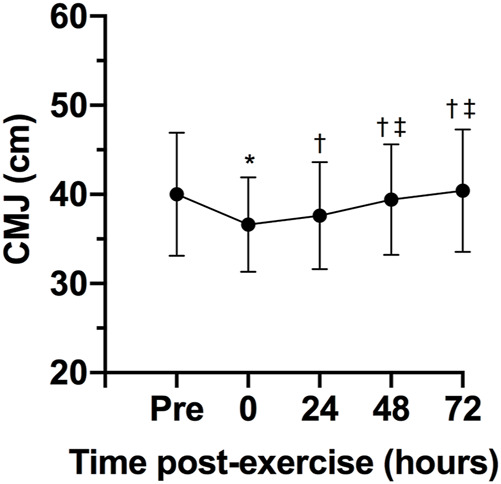
Variables are expressed as mean and standard deviation (±). Height of counter movement jump (CMJ) during pre-test, post-test, 24, 48 and 72 h post-test; *p ≤ 0.05 for pre-exercise; †*p* ≤ 0.05 for 0-h post-exercise; ‡*p* ≤ 0.05 for 24-h post-exercise.

There was a statistically significant effect of time on CK, F (4, 24) = 8.31, *p* < 0.0005, ES = 0.58, observed power = 0.99. The CK concentration 24 h after the exercise session was statistically significant higher that pre-exercise concentration (*p* = 0.040; Figure 3). No statistically significant differences were observed between 0- (*p* = 0.241) 48- (*p* = 0.608) and 72-h (*p* = 0.973) after exercise and pre-exercise concentrations. The Karen protocol had a statistically significant effect on CMJ, F (4, 28) = 4.14, *p* = 0.046, ES = 0.37, observed power = 0.59. The height of CMJ post-exercise was statistically significantly lower than pre-exercise (*p* = 0.043; Figure 4). However, no statistically significant differences were observed in the height of CMJ between pre-exercise and 24- (*p* = 0.108), 48- (*p* = 0.459) and 72-h (*p* = 0.827) post-exercise.

**FIGURE 5:**
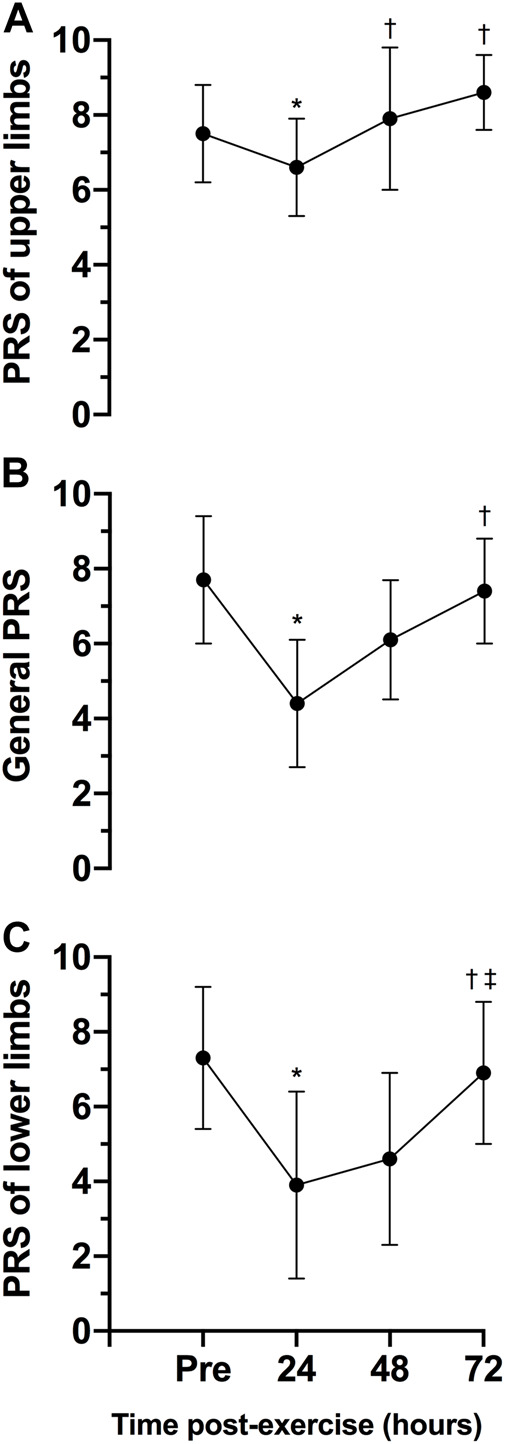
Variables are expressed as mean and standard deviation (±). Perceived recovery scale (PRS) of the upper limbs **(A)** general **(B)** and lower limbs **(C)** during pre-test, 24, 48 and 72 h posttest. *p ≤ 0.05 for pre-exercise; †*p* ≤ 0.05 for 24-h post-exercise; ‡*p* ≤ 0.05 for 48-h post-exercise.

Figure 5 shows the general, lower and upper limbs PRS of pre- and post-exercise session. There was a statistically significant effect of time on general PRS, F (3, 21) = 10.33, *p* < 0.0005, ES = 0.60, observed power = 0.98, lower limbs PRS, F (3, 21) = 739, *p* = 0.002, ES = 0.51, observed power = 0.96 and upper limbs PRS, F (3, 21) = 8.28, *p* = 0.001, ES = 0.54, observed power = 0.98. The scores of general, lower, and upper limbs PRS were statistically significant lower 24-h post-exercise session than pre-exercise (*p* = 0.013 for general, *p* = 0.037 for lower and 0.046 for upper limbs). No differences in the scores of PRS were observed between 48- (*p* = 0.647 for general, *p* = 0.244 for lower and *p* = 1.000 for upper limbs) and pre-exercise scores or between 72-h post-exercise (*p* = 1.000 for general, *p* = 1.000 for lower and *p* = 0.190 for upper limbs) and pre-exercise scores.

**FIGURE 6:**
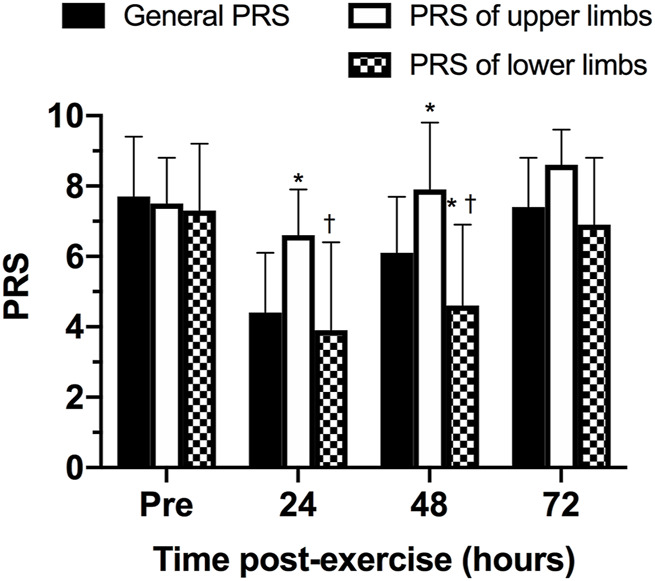
Comparison between scores of general, lower and upper limbs of perceived recovery scale (PRS) at different time points. *p ≤ 0.05 for general PRS; †*p* ≤ 0.05 for PRS of upper limbs.

The comparison between the scores of general, lower, and upper limbs of PRS was presented in Figure 6. No statistically significant differences were observed between PRS scales pre- (*p* = 1.000 between general and upper PRS scores; *p* = 0.262 between general and upper PRS scores; *p* = 1.000 between lower and upper PRS scores) and 72 h post-exercise (*p* = 0.107 between general and upper PRS scores; *p* = 0.332 between general and upper PRS scores; *p* = 0.093 between lower and upper PRS scores). However, 24- and 48-h post-exercise, the PRS of upper limbs was statistically significantly higher than general PRS (*p* = 0.015 for 24-h and *p* = 0.030 for 48-h) and PRS of lower limbs (*p* = 0.041 for 24-h and *p* = 0.014 for 48-h). Finally, 48-h post-exercise, the PRS of lower limbs was statistically significantly lower than general PRS (*p* = 0.037).

### Correlations

Table 2 shows the correlations between the PRS scales, CK concentration and height of the CMJ. It was observed only a statistically significant correlation between PRS of upper limbs and height of the CMJ (*p* < 0.0005; *r* = 0.533; large).

**TABLE 2:** Correlation of creatine kinase (CK) concentration, height of counter movement jump (CMJ) and perceived recovery scales (PRS).

	**CK Concentration (U/L)**	**Height of CMJ (cm)**
General PRS	*r* = -0.228	*r* = 0.184
	*p* = 0.242	*p* = 0.314
PRS of upper limbs	*r* = 0.075	*r* = 0.533*
	*p* = 0.705	*p* < 0.0005
PRS of lower limbs	*r* = -0.149	*r* = 0.007
	*p* = 0.450	*p* = 0.968

Note: Pearson correlation test. The magnitude of the correlations was classified as: r ≤ 0.1 trivial; 0.1 < *r* ≤ 0.3 small; 0.3 < *r* ≤ 0.5 moderate; 0.5 < *r* ≤ 0.7 large; 0.7 < *r* ≤ 0.9 very large; > 0.9 almost perfect. *p ≤ 0.05 for relationship between PRS, of upper limbs and height of CMJ.

## Discussion

The aim of this study was to analyze the physiological, biochemical, and neuromuscular responses following a CrossFit^®^ benchmark session and to assess if the PRS scale could be a practical tool to determine the athletes’ readiness to train status. The main findings partially confirm the initial hypothesis, revealing 1) significant increases in blood lactate post-exercise; 2) an increase of CK concentration 24 h post-exercise, returning to baseline levels 48 h post-exercise; 3) a significant change in the participants’ perceived recovery status PRS for upper and lower limbs 24 h post-exercise when compared to baseline, with PRS values for the lower and upper limbs showing different rates of recovery at 24- and 48-h post exercise (with the lower limbs’ PRS recovering slower than the PRS for the upper limbs). The findings corroborate previous studies that demonstrate the significant physiological, biochemical, and neuromuscular changes following a CrossFit^®^ session (Mate-Munoz et al., 2017; Gomes et al., 2020; Martinez-Gomez et al., 2022). Importantly, this study highlights the potential of the PRS scale to be used as a marker of recovery status following a Crossfit^®^ session.

CrossFit^®^ training sessions are often performed with near-maximal or maximal efforts, leading to a significant metabolic stimulus (Tibana et al., 2016; Claudino et al., 2018). In this context, blood lactate concentration has been utilized as a reliable marker to assess the intensity of different sessions of CrossFit^®^ (Falk Neto et al., 2020). While changes in blood lactate concentrations will be dependent on the duration and intensity of the sessions performed (Özsu et al., 2018), previous research has shown that different CrossFit^®^ sessions incur high blood lactate levels (Toledo et al., 2021). Timon et al. (2019) analyzed the blood lactate responses of two different protocols (Protocol 1: AMRAP of Burpees and Toes to Bar increasing repetitions (1–1, 2–2, 3–3 … ) in 5 minutes; Protocol 2: three rounds of 20 repetitions of wall ball (9 kg) and 20 repetitions of power clean (40% 1RM) in the shortest possible time), with protocol two showing a similar lactate response as the one seen in this study (18.38 ± 2.02 mmol/L vs. 17.5 mmol/L ±3.0 mmol/L). Despite a similar perception of effort, it seems that protocols that do not use an external load (protocol 1) have a smaller lactate response (Timon et al., 2019). Still, the metabolic response in these sessions is considered high, even in the absence of an external load. For example, a session requiring participants to complete as many rounds as possible (AMRAP) of two exercises (burpees and toes to bar) still elicited a high blood lactate response (13.3 ± 1.87 mmol/L). Tibana et al. (2016), analyzing a session that involved AMRAP of double under and rowing, and Maté-Muñoz et al. (2017) with a session that consisted of performing a single exercise (double unders), also reported a high lactate response (9.05 ± 2.56 vs. 10.37 ± 2.91 mmol/L), respectively. In addition, even when the intensity of a CrossFit^®^ session was manipulated to be performed at a lower perception of effort (6 out of 10, utilizing the Borg CR-10 scale), the lactate responses were still quite high (12.8 ± 3.2 mmol/L) (Alsamir Tibana et al., 2019). Previous studies have demonstrated that the metabolic responses induced by a training session are related to the required time to recover from this stimulus (Özsu et al., 2018). Considering the high physiological stress induced by CrossFit^®^ sessions, even when there is no external load, or when the intensity is controlled, understanding the time-course of recovery from these sessions is essential to ensure athletes can optimize their training.

The serum CK is often utilized to understand the recovery status of participants following a training session given its easy of collection and analysis (Halson, 2014). The CK concentrations can be raised due to exercise induced muscle damage as a consequence of intense and prolonged training. The peak of serum CK normally occurs about 12–24 h after a strength training session, and values can remain elevated for up to 96 h when the exercise is focused on the eccentric phase of the movement (Baird et al., 2012). Importantly, CK values have been associated with muscle injury (Hyatt and Clarkson, 1998; Halson, 2014). Studies involving CrossFit^®^ showed significant increases in CK that could be pathological due the extremely high values (Tibana et al., 2018b; Meyer et al., 2018). The present study found increases in CK 24 h post-exercise, with the values returning to baseline 48 h post-exercise. These results are in agreement with Timon et al. (2019) that evaluated the time course of recovery of CK in response to two different CrossFit^®^ WODs. Both sessions induced a significant increase in CK levels 24 h post-exercise, with the values decreasing and returning to baseline 48 h post-exercise. Similarly, Tibana et al. (2019) showed that after five workouts over three consecutive days of competition the peak CK concentration occurred 24 h post-exercise (∼698.7 U/L). Thus, it seems that when the CrossFit^®^ session does not elicit increases in CK concentration that could be considered pathological, the concentrations might return to baseline levels within 48 h.

In addition to changes in CK concentrations, CMJ height alterations might also be utilized as a potential marker of fatigue (Claudino et al., 2017). A recent study analyzed CMJ height as a measure to assess neuromuscular status following a CrossFit^®^ competition (Tibana et al., 2019). The CMJ jump height was significantly reduced 24-h post competition, with the values collected at 48- and 72-h post competition showing no differences from baseline. However, Tibana et al. (2016) demonstrated that consecutive days of CrossFit^®^ training, despite eliciting significant metabolic changes, did not lead to impairments in muscle power. Considering that CrossFit^®^ sessions vary often in the exercises performed and consequently, muscle groups utilized, and their duration, it is possible that CMJ height might have limited application as a measure to monitor the athletes’ neuromuscular status, particularly after single bouts of exercise.

The novel finding of this study is that while objective measures (CK and CMJ height) indicate that the participants might be fully recovered from a session within 24–48 h, the psychobiological monitoring of the athlete’s perceived recovery state indicates that 48–72 h might be needed for the athletes to return a point where performance is expected to be the similar or improved, based on the PRS. Psychobiological monitoring of training status is a non-invasive and non-exhaustive measure of assessing fitness (e. g. stress, fatigue), and also presents an effective and inexpensive measure to assess individual responses to training and competition. Despite its possibility as a tool to monitor current training status, Bishop et al. (2008) reported that there is still a limited knowledge by trainers and athletes about how to utilize such tools to optimize training intensity and recovery within a microcycle. Nevertheless, the large effect sizes reported indicate that further studies are required to assess the efficacy of the general PRS scale to determine the athletes’ recovery status. The different time course of recovery for the upper and lower limbs, with the perception of recovery for the lower limbs taking a longer time to return to baseline levels, has practical significance in CrossFit^®^. Coaches and practitioners can potentially use this information to prescribe the next training bout in a way that respects the recovery time required following the previous session. In this scenario, prescribing a training session that focuses on the upper or lower body, or controlling the intensity of the subsequent session might assist coaches in reducing the intensity of the subsequent session, when required, or to reduce the level of physiologic stress, consequently, properly managing the athlete’s training load (Tibana et al., 2018a; Falk Neto et al., 2020). While these would be important outcomes to ensure improved training prescription in the modality, further studies are required in this topic.

Despite a range of instruments to monitor recovery have been established, many are impractical for daily use due to cost, time, and challenges with interpretation (Lee et al., 2017; Seshadri et al., 2019). The results in this study demonstrate that a practical, non-invasive and expeditious approach to monitoring the participant’s recovery following an acute CrossFit^®^ session might provide important information for coaches and practitioners. In particular, the time-course of recovery according to the PRS is similar to that of the CK responses, with both measures reaching its most extreme values 24 h after the training session. However, while CK responses recover faster in the subsequent 24 h, the athletes’ perceived recovery might show a slower improvement, particularly for the lower limbs based on the protocol used in this study. Therefore, this study demonstrates that the PRS may be useful in allowing appropriate adjustments in training intensity or volume in CrossFit^®^ based on the athletes’ recovery status. Considering the potentially detrimental effects of performing numerous maximal or near-maximal CrossFit^®^ sessions in a short period of time, the use of the subsets of the PRS scale (upper and lower limbs) might assist in optimizing training prescription, providing important information about when the next stimulus should be provided, according to the athletes recovery status. Future studies should investigate if the use of the PRS scale might, in fact, optimize training prescription while helping to reduce the incidence of muscle injuries and the onset of non-functional overreaching.

Some limitations of the present study must be emphasized. Particularly, the reduced numbers of participants, the lack of control over the participants’ diet prior to the test must be acknowledged. In addition, other factors that could influence the participants’ recovery such as sleep, and stress have not been assessed during this study. Caution is advised when extrapolating the results of the current study to other populations or individuals of different training experience, as only healthy, experienced and male participants were recruited in this study. Our findings should not be generalized for other WOD and exercises. Moreover, our results cannot be used to infer the effects of combining these sessions within a larger training week, including a match stimulus and other modes of training (i.e., gymnastics, strength, power, and cardiorespiratory training). Future studies of a similar nature should include other critical biomarkers and an upper limb power measures to elucidate the time course of recovery and whether a state of fatigue truly occurred. Further longitudinal studies analyzing fatigue status and recovery in response to CrossFit^®^ training over several days using similar methods can be relevant to further our understanding of the performance changes, and fatigue and recovery markers in different subjects.

## Conclusion

In summary, a single CrossFit^®^ session using repeated wall-ball movements elicited a significant level of metabolic stress, along with an increase in CK levels in the 24-h after the exercise session. More importantly, the results showed the potential utility of the PRS scale as noninvasive tool for accurately monitoring recovery status in CrossFit^®^ practitioners. Particularly, the subscales of the PRS (upper and lower limb) seemed to be more effective at assessing changes in the athletes’ perceptions of recovery following an acute session. Coaches, sport scientists, and practitioners could implement the use of these scales PRS to obtain important insights into the recovery status of the participants. While this information can be useful to coaches to optimize training monitoring and to minimize the potential detrimental effects associated with the performance of repeated high-intensity sessions of CrossFit^®^, further studies are required to test this hypothesis.

## Data Availability

The raw data supporting the conclusion of this article will be made available by the authors, without undue reservation.
